# Webulous and the Webulous Google Add-On - a web service and application for ontology building from templates

**DOI:** 10.1186/s13326-016-0055-3

**Published:** 2016-04-01

**Authors:** Simon Jupp, Tony Burdett, Danielle Welter, Sirarat Sarntivijai, Helen Parkinson, James Malone

**Affiliations:** European Bioinformatics Institute (EMBL-EBI),European Molecular Biology Laboratory, Wellcome Trust Genome Campus, Hinxton, Cambridge, UK

**Keywords:** OWL, Ontology, Spreadsheet, Webulous, Google App

## Abstract

**Background:**

Authoring bio-ontologies is a task that has traditionally been undertaken by skilled experts trained in understanding complex languages such as the Web Ontology Language (OWL), in tools designed for such experts. As requests for new terms are made, the need for expert ontologists represents a bottleneck in the development process. Furthermore, the ability to rigorously enforce ontology design patterns in large, collaboratively developed ontologies is difficult with existing ontology authoring software.

**Description:**

We present Webulous, an application suite for supporting ontology creation by design patterns. Webulous provides infrastructure to specify templates for populating ontology design patterns that get transformed into OWL assertions in a target ontology. Webulous provides programmatic access to the template server and a client application has been developed for Google Sheets that allows templates to be loaded, populated and resubmitted to the Webulous server for processing.

**Conclusions:**

The development and delivery of ontologies to the community requires software support that goes beyond the ontology editor. Building ontologies by design patterns and providing simple mechanisms for the addition of new content helps reduce the overall cost and effort required to develop an ontology. The Webulous system provides support for this process and is used as part of the development of several ontologies at the European Bioinformatics Institute.

## Introduction

Like most data resources, ontologies are rarely complete, and healthy ontologies are continually growing and improving, as the state of knowledge progresses [1, 2]. Typically, authoring ontologies is a task performed by trained experts, familiar with ontology development practices and the complexities of languages such as the Web Ontology Language (OWL). This presents a major bottleneck to the ontology development process as the time and availability of trained experts is limited and ontology development is hard to fund [3]. Tools are now being developed to simplify the addition of content to ontologies that are based on populating ontology design patterns via data entry templates.

Ontology design patterns (ODPs) are commonly used in ontology development in guiding the ontology developer in the modeling of knowledge [4, 5]. They also help in enforcing consistency and best practice in ontology design whilst reducing arbitrary class descriptions within an ontology that can lead to both errors and ontologies that are difficult to maintain. Whilst ODPs can provide a sound methodological framework, ontology expertise is still required to establish and apply modeling patterns for real-world entities from a particular domain of interest [6].

Several tools have been previously developed to support building OWL ontologies from design pattern templates [1, 7, 8]. The main aim of these tools is to provide a simple interface for populating a design pattern that shields the users from the underlying OWL vocabulary. These systems help to enforce rigour and adherence to a design pattern and allow new content to be added in bulk in a reproducible manner. Although these tools help in enforcing consistency of ontology development, in order to truly mediate content contributions from non-ontology experts, tools that use a familiar paradigm to domain experts are required. Such tools should enable non-ontologists to contribute whilst also tackling the issues of translating input into OWL ontologies.

In this paper we describe the Webulous framework that provides software for the management of ontology design patterns and ontology building templates. Webulous is built around a client/server architecture, where the server hosts a number of ontology building templates that can be served to any number of client applications. Data submitted to the server is translated into OWL assertions according to design patterns expressed in the Ontology Pre-Processing Language (OPPL) [9]. We have developed a client application for Webulous using the Google Sheets Add-On framework that allows design pattern templates to be loaded into Google Sheets and submitted back to a Webulous server for processing. The Webulous client is aimed at domain experts adding new content to ontologies and is demonstrated as a term submission tool for the Experimental Factor Ontology [10].

## Results

Webulous provides a public service for the creation and management of ontology design templates. A Webulous server can host a number of ontology building templates that use OPPL statements to translate input data into OWL axioms. A Webulous template specifies a series of fields for the input data, and fields can can be restricted to only allow values from a list of ontology terms. The Webulous API can be used by client side applications to automatically build the user interface for a given template. Once a user populates a template with data this is submitted back to a Webulous server where the patterns are instantiated to create new OWL statements ready for import into the target ontology.

### Google Sheets Add-on

Providing Webulous as a service means that a range of client-side applications can be developed for populating a template. We built a Google Sheets Add-On that supports loading Webulous templates from a server and submitting populated templates back to the server for processing. We chose Google Sheets for their convenient document management and sharing functionality and for the familiarity of the spreadsheet format for users.

When a Webulous template is loaded via the Google Add-On, each template input field represents a column in the sheet. Columns can be restricted to a set of allowed ontology terms by using term labels to create data validation. This data validation provides the user with convenient term autocomplete when entering data into a cell and will alert the user when an invalid term has been entered. Data submitted from Google Sheets is associated with the user’s Google account so the server can notify both the user and template admin via e-mail once the template has been processed.

**Fig. 1:**
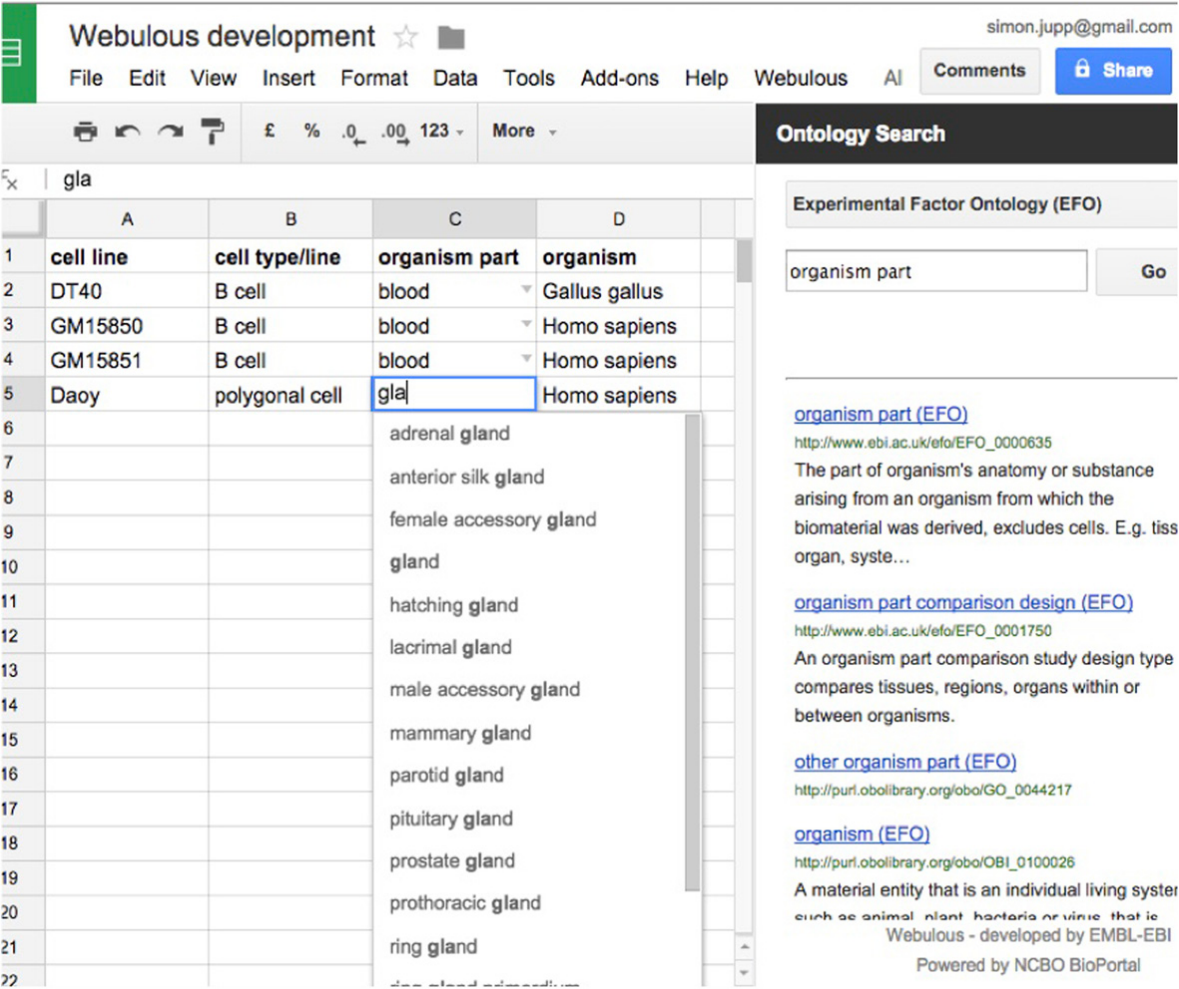
Webulous Google Add-on. Screenshot of the Webulous Google Sheets Add-on showing a ontology-based data validation

The Webulous Google Sheets Add-On (Fig. 1) has additional functionality by allowing users to connect directly to BioPortal services [11]. The Webulous Add-on provides a side bar for searching BioPortal for ontology terms and creating custom ontology-based data validations. The sidebar allows users to create a validation, which consists of a restricted set of term labels, and provides a convenient way to create further validations using subclasses of any particular term.

### Application of Webulous

The Experimental Factor Ontology contains descriptions of experimental variables ranging from diseases, cell types, cell lines, anatomy, assays, chemical compounds and phenotypes. It is developed as an application ontology that integrates and bridges several external reference ontologies (such as ChEBI and the Gene Ontology). EFO enriches these existing ontologies by including additional axioms that connect terms like diseases to tissues and anatomical systems; cell lines to cell types, diseases and tissue; and link common and rare diseases through associated anatomical parts and phenotypes. EFO is used to annotate resources spanning multiple omics including; transcriptomics data in ArrayExpress [12] and Expression Atlas [13], genomics data in the NHGRI-EBI GWAS Catalog [14], proteomics data in PRIDE [15] and cell line data in Encode [16]. EFO is also used by the Centre for Therapeutic Target Validation (CTTV)^1^ as their core data annotation resource.

One of the appealing features of EFO is that many of the design patterns are well established and applied consistently across large portions of the ontology. This use of design patterns makes EFO nicely amenable to the generation of new content using templates. Prior to the work presented here most cell lines have been added to EFO for the ENCODE project using Excel-based spreadsheets that were processed with Populous [17]. As more resources adopt EFO there is an increasing pressure on the editors to add new content, much of which remains in a spreadsheet-based format on submission.

A dedicated Webulous instance is now running at the EBI to serve templates for adding new content to EFO^2^. This instance currently contains six EFO templates summarised in Table 1. Four of these templates are for adding new terms to EFO that include new cell lines, diseases, assays or measurement terms. There is a dedicated template for adding synonyms to existing terms and a more general template for adding other types of annotation properties such as external cross-references.

**Table 1:** Summary of EBI Webulous templates

Template name	Number of fields	Number of design patterns
Add EFO cell Line	9	7
Add EFO synonyms	2	1
Add EFO assay	7	6
Add EFO disease	19	18
Edit EFO annotation	2	1
Add EFO measurement	14	13

Users load these templates directly from the Google Sheets Webulous Add-On by simply connecting to the Webulous server running at the EBI. Once a pattern is selected a spreadsheet-based template will be created in the Google Sheet. Figure 1 shows the Cell line pattern loaded into a Google Sheet. It also shows how the data restrictions on fields have been used to create data validations on some of the columns to assist the user in data entry.

**Fig. 2:**
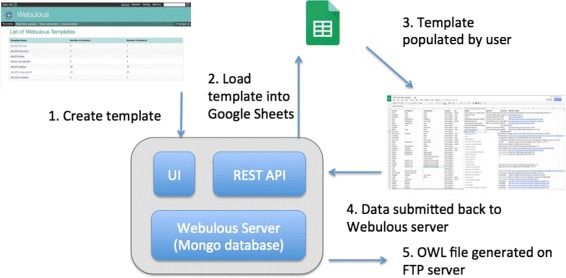
Webulous architecture. The typical data flow for creating ontology content from Webulous and the Google Add-On. Templates are created using the Webulous user interface and are made available to client application via a REST API. The Google Add-On can be configured to load templates from any public Webulous server. Once a template has been populated by a user, they can submit this data back to the Webulous server for processing. Webulous will process the data to generate an OWL file and notify the user once complete with a link to the newly generated OWL file

Once data is submitted by a user from the Google Sheet, it gets processed on the Webulous server to generate an output file containing the newly generated axioms. Both the submitter and EFO curators are notified via e-mail if the submission is either successful or has failed. In cases of failure due to, e.g., missing information or an unsatisfiable class inadvertently created by a user, EFO curators may go back to the submitter to fix the issue in the source sheet. The ability to easily share documents via Google Sheets means that EFO editors and submitters can work collaboratively on the submission. Once a submission is successful, EFO editors can open the OWL file generated by Webulous in Protégé alongside the latest source file to inspect the changes. The new content is both manually validated by EFO editors, and a series of automated ontology validation scripts that check for common errors such as duplicate labels or definitions are executed. Finally the newly generated axioms are merged into the release candidate and the URIGen Protégé plugin^3^ is used to assign new EFO URIs where applicable. Figure 2 shows the Webulous architecture and how data flows between the Webulous server and the Google Add-On.

The EFO Webulous server has been running and accepting submission via this route since April 2015. By December 2015 EFO had received over 20 data submissions that each included batches of new term requests or the addition of term annotations. The data submissions and the generated output files can be viewed at http://www.ebi.ac.uk/efo/webulous/submissions. Webulous is being used by both EFO core developers and by external database curators from the Gene Expression Atlas, COSMIC and UniProt databases. Table 2 summarises the Webulous generated content in EFO as of EFO version 2.65. In total 1479 new terms were created via the Webulous route and a total of 13,133 new axioms generated.

**Table 2:** Summary of EFO content generated via Webulous data submissions

	Classes	Axioms	Submissions
ATLAS - cell lines	566	3256	15
ATLAS - diseases	113	511	3
COSMIC - cancers	800	3707	1
UniProt - OMIM xRef	0	5659	1
Total (as of EFO 2.65)	1479	13133	20

### Availability

A public Webulous server is currently being hosted by EMBL-EBI at http://www.ebi.ac.uk/spot/webulous, where users can create their own custom templates and access them from Google Sheets. Data submitted to the EBI server is processed on the EBI Load Sharing Facility (LSF) computing cluster to provide highly scalable infrastructure for executing OPPL patterns over large ontologies. The Webulous Service for submitting EFO terms is available at http://www.ebi.ac.uk/efo/webulous. The Webulous Google Sheets Add-on is available to install form the Google Chrome store at https://goo.gl/KoHA8k. Webulous is open source and the code is kindly hosted by GitHub at https://github.com/EBISPOT/webulous.

## Method

Webulous provides a client-server architecture for the both the management of design patterns and the transformation of data to OWL axioms according to a set of applied patterns. Patterns are expressed in the OPPL language and the Java OPPL API is used to process data into OWL. For example, in OPPL we can define a simple design pattern for modeling cell nucleation as follows:

*Example 1: OPPL pattern for cells and nucleation*?cell:CLASS,?nucleation:CLASSBEGINADD ?cell SubClassOf hasNucleation some ?nucleationEND;

This pattern defines two variables, *?cellType* and *?nucleation*, that are typed as OWL classes, and an OWL subclass axiom that represent the cell nucleation design pattern. By assigning concrete classes for cell type and nucleation, such as *blood cell* and *anucleate*, the OPPL API could be used to generate new OWL axioms.

**Fig. 3:**
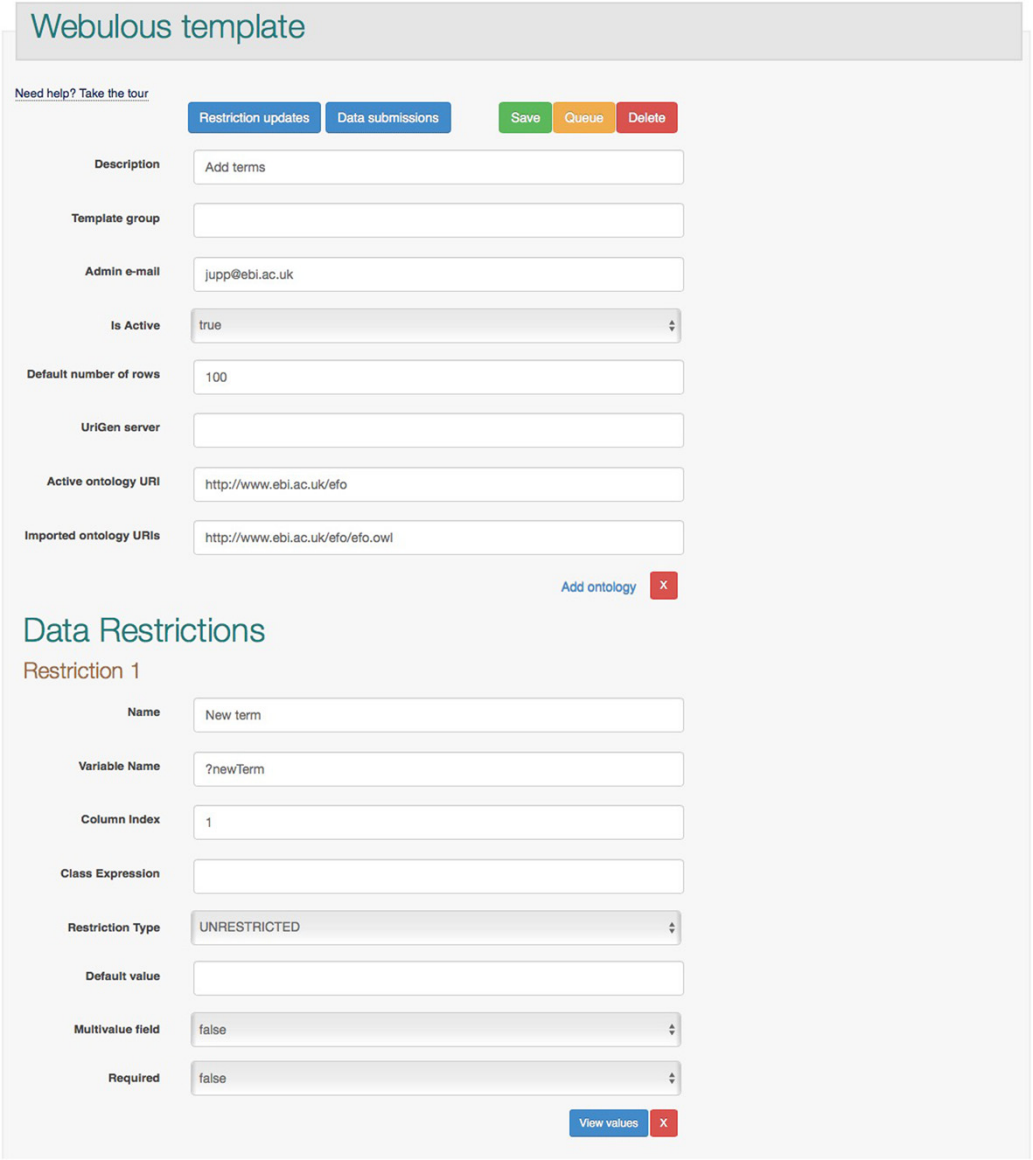
Webulous template server. Screenshot of the Webulous template administration interface showing the fields needed to describe a new template

A Webulous template (Fig. 3) must include at least one input field. Templates can specify if an input field accepts free text data (used for capturing literal data types) or is restricted to a set of pre-existing ontology terms. The list of terms used by template can be a custom list or it can be generated dynamically using description logic (DL) queries across one or more ontologies associated with the template. Webulous will automatically update the template when new releases of those ontologies become available, ensuring client applications are always working with the latest version of the ontologies.

**Fig. 4:**
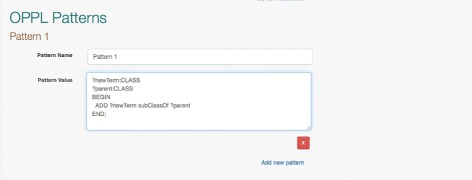
OPPL design patterns in Webulous Screenshot of the Webulous template administration interface showing a design pattern expressed in OPPL

Each template can have one or more design patterns (Fig. 4) associated with it that will be executed with data submitted from a client application. Design patterns expressed in OPPL support an almost complete set of OWL 2 constructs and can be used to generate T-box (class level), A-box (instance level) or non-logic based annotation assertions. The expressivity afforded by OPPL means that Webulous could be used for building both OWL ontologies and RDF knowledgebases.

Webulous works by linking fields in the template to variables in the OPPL patterns. Consider the following template that could be used to add new terms to an existing ontology. For this template we want to define a field for the new term, a field for the parent class and a field for a term definition.

Using Webulous we would create the following:

1. Create a new template called “Add terms”.
2. Add the source ontology as an imported ontology.
3. Create three data restrictions, one for each input field
  - The first field is where we want users to input the new term name. We call this field “New term” and assign it to a variable called *?newTerm*
  - The second input field will be the parent class of the new term. We call this field *?parent* and we want to restrict the valid entries to any term in the source ontology. We do this by specifying the DL query as *owl:Thing* and selecting the descendants option. We assign the field to the *?parent* variable.
  - Finally we want a field where the user can enter a textual definition. We call this field “definition” and leave it as an unrestricted field with the variable name *?definition*.
4. We can use two OPPL patterns to transform any input data to OWL
  - Pattern 1 is used to create a subclass relation between the new newly created class in field 1 and the named class in field 2. The OPPL pattern uses the variable names to refer to the input fields.
    ?newTerm:CLASS
    ?parent:CLASS
    BEGIN
    ADD ?newTerm subClassOf ?parent
    END;
  - Pattern 2 is used to create an annotation assertion between the newly created class in field 1 and the textual definition supplied in field 3.
    ?newTerm:CLASS
    ?definition:CONSTANT
    BEGIN
    ADD ?newTerm.IRI definition ?definition
    END;
5. On saving this template Webulous will load the source ontology in order to prepopulate the list of allowed values in field 2 so that it is ready to serve the template to a client application.

This template is then ready for loading into a client application for user input. Once the user has populated the template with data, this can be submitted back to the Webulous server for processing and conversion to OWL. The Webulous server processes a submission by taking each row in the input data and applying the OPPL patterns associated with the template. Once all the data has been processed by OPPL, the axioms are collected together and written to a single file and made available on the Webulous FTP server^4^.

Webulous templates can be further configured to specify if fields are mandatory or optional. Users can enter data using the primary label rather than id for fields that are restricted to existing ontology terms. As Webulous is primarily aimed at generating new ontology content, any value that is not recognised by label in the source ontology will be created as a new term in the ontology. If the label already exists, Webulous will use the URI for that term, making it possible to refer to existing terms in an ontology. By default a random URI will be generated for the term with the user entered value set as the label. Webulous can be configured to create new URIs according to an incremental id strategy or can connect to a URIGen server for minting new term URIs.

### Webulous implementation

The Webulous server is built in Java and includes an embedded Apache Tomcat^5^ server so it can be run directly or deployed in any other Java servlet container. The primary database for storing templates is MongoDB^6^ and the Spring Data and MVC^7^ frameworks are used to provide the REST API. Webulous includes a series of scripts for updating templates and processing data submissions that can be run as part of scheduled job. Using the scripts means that CPU and memory-intensive tasks such as executing DL queries and OPPL scripts are run outside the web server so as to avoid memory bottleneck issues with the web service. The OPPL patterns are processed with the OPPL 2 Java API^8^ and further processing done using the Java OWL API. OPPL2 uses the HermiT [18] reasoner for querying the target ontology.

## Discussion

Previous work in developing tools for ontology authoring by domain experts generally have two components; a data input component that is aimed at the user, and a data transformation component that is responsible for transforming the user input into OWL axioms. TermGenie [1] is the primary application for adding new terms to the Gene Ontology [19]. TermGenie allows users to create new terms using formally specified design patterns. TermGenie provides a simple form-based interface for each template that can have restrictions on the set of terms allowed for a particular inout field. The input data is transformed into OWL axioms on the server and validated using an OWL reasoner. TermGenie uses an XML and JavaScript-based system for configuring new templates and has been demonstrated for use with other OBO foundry ontologies [20]. One of the limitations of TermGenie is that it is tightly coupled to the development of OBO foundry ontologies, so is therefore not a generic tool for ontology building via templates.

RightField [21] and OntoMaton [22] are examples of ontology-aware data input tools. They are aimed at creating spreadsheet-based templates where regions of the spreadsheet are restricted to values from a list of ontology terms. Spreadsheets are a popular data entry tool and have the benefit of being both familiar to user and support the input of data in bulk. OntoMaton is built as an Add-On to Google Sheets, so it has the added benefits of the collaborative support offered by Google Documents^9^. However, neither tool provides support for transforming the input data into OWL axioms.

Whilst data can be readily transformed to OWL using APIs such as the OWL API [23], there is an increasing demand for domain specific languages (DSLs) for working with OWL that are decoupled from any particular programming language and provide a more abstract representation of the design pattern. The Manchester OWL syntax [24] was designed as a more user-friendly syntax for expressing OWL constructs and as such provides a good basis for a DSL. Implementations of DSLs based on the Manchester OWL syntax that can be used for ontology design patterns include the Ontology Pre Processing Language (OPPL) [25] and M2 [7]. Both OPPL and M2 are designed to support a form of Manchester OWL syntax that uses variables that can be assigned to values to form new OWL axioms.

Dedicated applications like MappingMaster [7] and OntoRat [8] were developed to support the conversion of data from spreadsheets into OWL, but they don’t provide support for the management and creation of the data entry templates. Populous [17] was developed as an extension to RightField to provide support for both the template creation and the data transformation in a single application. Populous uses the RightField component to create Microsoft Excel templates and extends this with support for transforming populated templates into OWL using OPPL. The Populous application demonstrated how Excel spreadsheets provided a familiar user-interface for users that could be used to populate ontology templates en masse. Populous has been used in the development of several ontologies [26], including the Experimental Factor Ontology (EFO).

The OPPL language is extremely powerful but the lack of support and documentation for OPPL makes writing new design pattern difficult. Another limitation is that OPPL currently requires the HermiT OWL reasoner. HermiT is a DL reasoner and it cannot classify many of the ontologies available in the life science in a reasonable time, even when run on large computing clusters with lots of allocated memory and CPU. We are currently investigating the use of different reasoners with OPPL, in particular highly-scalable EL reasoners, like ELK^10^. The Webulous system has been designed to support different types of DSLs, other than OPPL, so could be readily extended in the future to support others OWL pattern languages such as Tawny-OWL or DOS-DP^11^.

Feedback from users of the Webulous Google Add-On has been positive and users are happy to submit terms to EFO via this method. However, we have found that creating large data-validations of ontology terms in Google Sheets causes some performance issues. To retain performance within acceptable limits we have restricted the content of input fields to primary labels and avoid creating validations of over 5000 terms. In future we would like to perform expanded search over synonyms in the Google Sheet and we are therefore exploring ways to support server side lookups of terms and validation using Google Sheets. We are also planning to develop standalone JavaScript widgets that will build a template in any webpage. This type of client is targeted at database curation systems where curators often need to submit new term requests at the point of data annotation and will improve usability further.

## Conclusion

We have presented the Webulous Service along with a client application that runs as a Google Sheets Add-On. Webulous has been developed to provide domain neutral support for building ontology by design patterns. Webulous is being used in the development of ontologies at EMBL-EBI and is proving to be a successful service for the bulk submission of term requests by our users. Webulous is now the new primary submission route for a range of terms in EFO, including submission of new cell lines for databases such as BioSamples and projects such as Encode. New disease-to-phenotype bridging axioms are being generated using Webulous as part of the Centre for Therapeutic Target Validation (CTTV) knowledge base. Webulous is also being used for the development for the Cellular Microscopy Phenotype Ontology (CMPO)^12^, an ontology being developed to annotate several cellular imaging databases including the BBSRC Image Data Repository (IDR).

Webulous is designed to complement existing development strategies and free up the time expert ontologists spend manually inserting new terms and to allow expert users of ontologies to perform knowledge representation directly on spreadsheets rather than using tools such as Protégé. The move to building the ontology by design patterns means that we can apply more rigour to ontology development. This kind of automation is especially important when an ontology grows to a size where human curators can no longer evaluate the content of the ontology as a whole before each release.

A range of tools are required to support large-scale ontology development projects, and Webulous is designed to support a specific scenario where domain experts wish to submit new ontology terms following well-established design patterns. Applications like TermGenie have shown that this approach can be successful for individual term requests, and Webulous extends this to provide an alternative interface that supports batch submissions and is configurable for any ontology.

## Endnotes

^1^https://www.targetvalidation.org.

^2^http://www.ebi.ac.uk/efo/webulous.

^3^https://github.com/simonjupp/urigen.

^4^ftp://ftp.ebi.ac.uk/pub/databases/spot/webulous/efo.

^5^http://tomcat.apache.org.

^6^https://www.mongodb.org.

^7^http://projects.spring.io/spring-data/.

^8^http://oppl2.sourceforge.net.

^9^https://www.google.co.uk/docs/about.

^10^https://code.google.com/p/elk-reasoner/.

^11^https://github.com/dosumis/dead_simple_owl_design_patterns.

^12^http://www.ebi.ac.uk/cmpo.
